# Preoperative predictors for early recurrence of resectable pancreatic cancer

**DOI:** 10.1186/s12957-016-1078-z

**Published:** 2017-01-10

**Authors:** Kohei Nishio, Kenjiro Kimura, Ryosuke Amano, Sadaaki Yamazoe, Go Ohrira, Bunzo Nakata, Kosei Hirakawa, Masaichi Ohira

**Affiliations:** 1 Department of Surgical Oncology, Osaka City University Graduate School of Medicine, 1-4-3 Asahimachi, Abeno-ku, Osaka 545-8585 Japan; 2 Department of Surgery, Kashiwara Municipal Hospital, 1-7-9 Hozenji, Kashiwara City, Osaka 582-0005 Japan

**Keywords:** Resectable pancreatic cancer, Preoperative predictors, CA19-9, Histological grade, Tumor diameter

## Abstract

**Background:**

The first-line treatment for resectable pancreatic cancer (RPC) is surgical resection. However, our patients have often experienced early recurrence after curative resection for RPC, with desperately poor prognosis. Some reports indicated that minimally distant metastasis not detected at operation might cause early recurrence. The present study aimed to identify preoperative clinicopathological features of early recurrence after curative resection of RPC.

**Methods:**

Ninety RPC patients who underwent curative resection between 2000 and 2014 at our institution were retrospectively analyzed.

**Results:**

Of the 90 patients, 32 had recurrence within 1 year. Univariate analysis demonstrated that preoperative serum carbohydrate antigen (CA19-9) ≥529 U/mL (*P* = 0.0011), preoperative serum s-pancreas-1 antigen (SPan-1) ≥37 U/mL (*P* = 0.0038), and histological grades G2–G4 (*P* = 0.0158) were significantly associated with recurrence within 1 year after curative resection. Multivariate analysis demonstrated that preoperative serum CA19-9 ≥ 529 U/mL (*P* = 0.0477) and histological grade G2–G4 (*P* = 0.0129) were independent predictors of recurrence within 1 year. Recurrent cases within 1 year postoperatively had significantly more distant metastasis than cases with no recurrence within 1 year (*P* < 0.001).

**Conclusions:**

Preoperative serum CA19-9 ≥ 529 U/mL and histological grades G2–G4 were independent predictive factors for recurrence within 1 year after pancreatectomy for RPC. Furthermore, recurrent cases within 1 year had more frequent distant metastasis than cases with no recurrence within 1 year. These results suggest that RPC patients with preoperative serum CA19-9 ≥ 529 U/mL should receive preoperative therapy rather than surgery.

## Background

Patients with pancreatic cancer have been reported to have a 5-year overall survival (OS) of approximately 5% [1]. Only 20% of patients with pancreatic cancer are candidates for potentially radical resection [2]. Surgical resection is associated with a median OS of 11 to 23 months and with a 5-year OS of about 20% [3, 4]. Furthermore, 60% of patients experience local and systemic relapse within the first 12 months after curative surgery [3]. Preoperative predictors of survival time after surgery have been reported as tumor size [3], preoperative lymph node metastasis [3], preoperative serum carbohydrate antigen (CA19-9) level [2, 3, 5], G3–G4 on pathological grading [2], duration of symptoms [2], and preoperative modified Glasgow Prognostic Score (mGPS) [6].

Moreover, some studies have suggested that minimally distant metastasis undetectable even by recent advanced diagnostic imaging might have existed at the time of surgery in patients with early recurrence [7, 8]. In fact, occult peritoneal or liver metastatic disease has been reported to be missed by CT in 4 to 15% of patients [3].

The National Comprehensive Cancer Network (NCCN) guidelines recommend surgical resection as the first choice for the treatment of RPC. However, some researchers found that median OS of patients with RPC was approximately 12 months after surgery alone [9]. Other researchers found that the median OS of patients with RPC was <2 years after surgery and adjuvant therapy [10, 11]. Furthermore, at least 25% of patients cannot receive adjuvant chemotherapy because of complications related to surgery [9]. One group of investigators found that the median OS in patients undergoing resection for RPC was 27 months using preoperative chemoradiation therapy, while the median OS was 17 months in patients receiving surgery alone (*P* = 0.004) [12]. However, no randomized controlled trials have been reported on the efficacy of neoadjuvant chemotherapy for RPC.

Although randomized controlled trials will give us the answer whether preoperative therapy is needed for all RPC, we think preoperative treatment is not needed for all RPC. If early recurrence of RPC can be predicted before performing surgery, we suppose that preoperative therapy should be selected prior to surgical resection for RPC patients.

The aim of this study was to identify preoperative predictors of early recurrence after curative resection for RPC. The results of this study could aid oncologists and surgeons in discriminating high-risk cases of RPC in which the cancer could recur within 1 year and selecting the patients who would benefit from preoperative therapy.

## Methods

### Study selection and inclusion and exclusion criteria

From January 2000 to December 2014, 183 patients underwent pancreatic resection for pancreatic ductal carcinoma at our institution. The study was approved by the ethics committee of Osaka City University and was in compliance with Helsinki declaration. Informed consent was obtained from all patients to use specimens for this study according to the institutional rules of our hospital. All patients were histologically confirmed to have the common type of invasive ductal carcinoma of the pancreas double checked by two pathologists. Any patients with neuroendocrine carcinoma, mucinous cystic carcinoma, or intraductal papillary mucinous carcinoma were excluded. Of the 183 patients, 93 patients were excluded for the following reasons: cases with R2 resection margins, 19; borderline resectable cancer, 52; unresectable cancer, 18; and censored cases, 4. The data from the remaining 90 patients were retrospectively analyzed. All of the 90 study patients were diagnosed with resectable pancreatic cancer according to the NCCN guidelines, version 2.2014.

### Outcome measures

The demographic and clinical variables included age, sex, body mass index (BMI), tumor location, tumor size, histological grade, preoperative serum s-pancreas-1 antigen (SPan-1) level, preoperative serum CA19-9 level, preoperative serum carcinoembryonic antigen (CEA) level, preoperative serum albumin, preoperative serum white blood count (WBC), preoperative serum lymphocyte count, preoperative serum C-reactive protein (CRP), and preoperative mGPS. In patients with preoperative jaundice, the data after the jaundice was reduced was used for the preoperative serum CA19-9 level. In patients with jaundice at our medical center, endoscopic or percutaneous bile duct drainage is usually performed. The CA19-9 level in all patients was the value after total bilirubin was reduced to <5 mg/dL.

### Surgery and pathology

Surgery involved standard or subtotal stomach-preserving pancreaticoduodenectomy in 41 patients (45.6%), distal pancreatectomy in 47 (52.2%), and total pancreatectomy in two (2.2%). Regional lymph node dissection was performed in all patients. The resected specimens were fixed in 10% formalin at room temperature, and the size and gross appearance of the tumor were recorded. The pathologic stage of all tumor specimens was determined using the American Joint Committee on Cancer, 6th edition, staging system [13]. Tumor differentiation was classified according to the World Health Organization (WHO) classification as well differentiated (G1), moderately differentiated (G2), poorly differentiated (G3), and undifferentiated (G4) [14].

### Adjuvant therapy and follow-up

All patients were followed for survival. Enhanced CT was performed every 4 months within 1 year after the surgery. One year later after the surgery, we performed enhanced CT every 6 months. If it was necessary, we added MRI or PET-CT. Basically before 2006, adjuvant therapy has not been performed. As adjuvant chemotherapy, gemcitabine was administered by reference to the results of CONKO-001 since 2006 [11]. S-1 was administered by reference to the results of JASPAC01 since 2013 [15].

### Statistical analysis

The clinicopathological features were compared between patients who experienced recurrence within 1 year (REC1) and those who did not (non-REC1). Categorical variables were compared using the *χ* ^2^ test or Fisher’s exact test. A receiver operating characteristic (ROC) curve was constructed to estimate the optimal cutoff value of preoperative serum CA19-9 level, preoperative serum SPan-1 level, tumor diameter, preoperative serum CEA level, WBC, lymphocyte count, and CRP. The cutoff value was determined as the point closest to the upper left-hand corner of the graph. Variables with a significance of *P* < 0.05 on univariate analysis were included in multivariate regression analysis to identify factors associated with recurrence within 1 year after surgery. Survival was calculated using the Kaplan-Meier method and compared between groups by the log-rank test. *P* values <0.05 were considered significant. On multiple comparison test, Bonferroni correction was used. Statistical analyses were performed using SAS version 9.0 software (SAS Institute, Inc., Cary, NC, USA).

## Results

### Characteristics of RPC patients

The characteristics of the 90 study patients who underwent curative surgery for RPC are given in Table 1. Among all the patients, 71 received adjuvant chemotherapy. The median follow-up period was 26.8 (5.6–175.2) months. The median OS was 41.5 months. The actuarial 3- and 5-year survival rates were 54.2 and 38.9%, respectively. The median preoperative serum CA19-9 level of the 90 patients was 92 U/mL. A ROC curve demonstrated that preoperative serum CA19-9 level of 529 U/mL was the optimal cutoff point for recurrence within 1 year after surgery, with sensitivity of 86.2% and specificity of 50%. The area under the curve (AUC) was 0.6815.

**Table 1:** Characteristics of patients with RPC

Characteristic	Number
Age	
Mean (range)	69.2 (34-88)
Sex	
Male	42
Female	48
BMI	
<17	7
18–25	67
>25	16
Location	
Head	42
Body-tail	48
Tumor size (cm)	
Mean (range)	2.8 (0.6-12)
Surgery	
Pancreaticoduodenectomy	41
Distal pancreatectomy	47
Total pancreatectomy	2
Histological differentiation	
G1	14
G2	63
G3	9
G4	4
UICC stage, Union for International Cancer Control	
IA	6
IB	11
IIA	31
IIB	39
III	1
IV	2
Lymph node	
N0	50
N1	40
Resection status	
R0	74
R1	16
Adjuvant therapy	
Yes	71
No	19

### Univariate and multivariate analyses of recurrence within 1 year after surgery

Of the 90 patients, 32 (35.6%) experienced recurrence within 1 year after surgery and 58 (64.4%) did not have recurrence within 1 year. The median OS of REC1 was 14.2 months, compared to the survival not reaching the median time in non-REC1, with a significant difference between the groups (*P* < 0.0001).

**Fig. 1:**
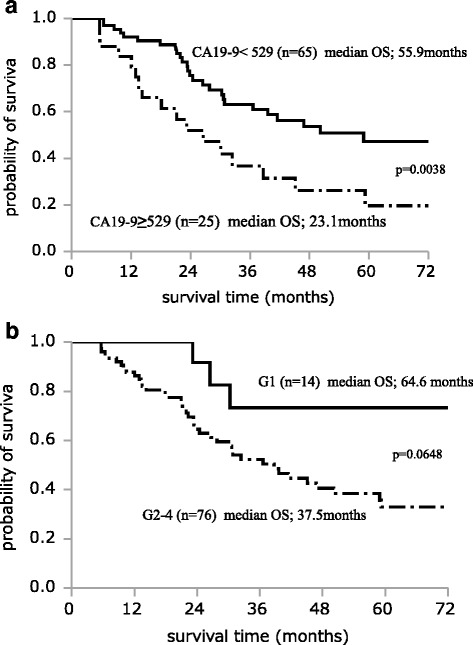
Survival curve of each independent risk factor. The median overall survival (OS) was 23.1 months in patients with CA19-9 ≥ 529 U/mL, compared to 55.9 months in patients with CA19-9 < 529 U/mL, with a significant difference between the groups (*P* = 0.0038) (**a**). The median OS was 37.5 months in patients with histological grades G2–G4, compared to 64.6 months in patients with G1, with no significant difference (*P* = 0.0648) (**b**)

**Fig. 2:**
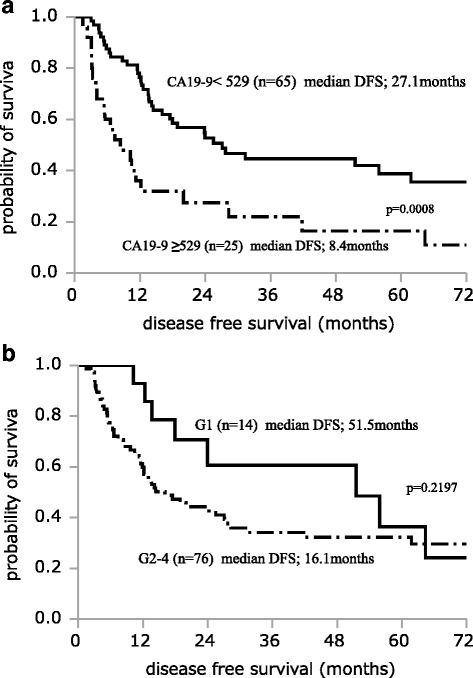
Disease-free survival of each independent risk factor. The median disease-free survival (DFS) was 8.4 months in patients with CA19-9 ≥ 529 U/mL, compared to 27.1 months in patients with CA19-9 < 529 U/mL, with a significant difference between the groups (*P* = 0.0008) (**a**). The median DFS was 16.1 months in patients with histological grades G2-G4, compared to 51.5 months in patients with G1, with no significant difference (*P* = 0.2197) (**b**)

**Fig. 3:**
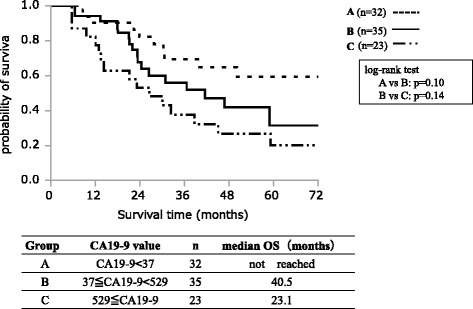
Survival curve of three groups stratified for preoperative serum CA19-9 level. The median OS in group A did not reach the median time, the median OS in group B was 40.5 months, and the median OS in group C was 23.1 months. The survival times were not statistically significantly different between the three groups, but stratified Kaplan-Meier curves could be drawn for these divided groups

Table 2 shows the results of univariate analysis of the factors affecting REC1 among the 90 study patients. Significant associations with REC1 were observed for histological grades G2–G4 (*P* = 0.0158), preoperative serum SPan-1 level ≥35 U/mL (*P* = 0.0038), and preoperative CA19-9 level ≥529 U/mL (*P* = 0.0011). Table 3 shows the results of multivariate analysis of factors affecting REC1 for RPC. Histological grades G2–G4 (OR, 8.906; 95% CI, 1.493–172.463; *P* = 0.0129) and preoperative CA19-9 level ≥529 U/mL (OR, 3.130; 95% CI, 1.012-10.132; *P* = 0.0477) were independent risk factors for REC1. Figure 1 shows the survival curves of each independent risk factor for REC1. The MST was 23.1 months in patients with CA19-9 ≥ 529 U/mL, compared to 55.9 months in patients with CA19-9 < 529 U/mL, with a significant difference between the groups (*P* = 0.0038). The median OS was 37.5 months in histological grade G2-G4, compared to 64.6 months in G1 (*P* = 0.0648). Figure 2 shows the disease-free survival (DFS) of each independent risk factor for REC1. Median DFS was 8.4 months in patients with CA19-9 ≥ 529 U/mL, compared to 27.1 months in patients with CA19-9 < 529 U/mL, with a significant difference between the groups (*P* = 0.0008). Median DFS was 16.1 months in patients with histological grade G2-G4, compared to 51.5 months in patients with G1, with no significant difference (*P* = 0.2197). In a further step, the 90 RPC patients were stratified into three groups: Group A, CA19-9 < 37 U/mL; Group B, CA19-9 ≥ 37 U/mL and <529 U/mL; Group C, CA19-9 ≥ 529 U/mL. We drew a survival curve for each group, as shown in Fig. 3. The median OS in Group A did not reach the median time, the median OS in Group B was 40.5 months, and the median OS in Group C was 23.1 months. The survival times were not statistically significantly different between the three groups, but stratified Kaplan-Meier curves could be drawn for these divided groups.

**Table 2:** Univariate analysis of factors affecting recurrence within 1 year after curative pancreatectomy for RPC

Characteristics	REC1 (*n* = 32)^b^	Non-REC1 (*n* = 58)^c^	*P* value
Age			0.6209
≥65	25 (78.1%)	42 (72.4%)	
<65	7 (21.9%)	16 (27.6%)	
Sex			0.3859
Male	17 (53.1%)	25 (43.1%)	
Female	15 (46.9%)	33 (56.9%)	
BMI			0.86
<17	3 (9.4%)	4 (6.9%)	
18–25	24 (75%)	43 (74.1%)	
>25	5 (15.6%)	11 (19%)	
Location			0.27
Head	12 (37.5%)	30 (51.7%)	
Body-tail	20 (62.5%)	28 (48.3%)	
Tumor size^a^			1
≥25 mm	19 (56.4%)	34 (58.6%)	
<25 mm	13 (43.6%)	24 (41.4%)	
Histological grade			0.0158
G2–G4	31 (96.9%)	45 (77.6%)	
G1	1 (3.1%)	13 (22.4%)	
Preoperative SPan-1^a^			0.0038
≥35	24 (75%)	24 (41.4%)	
<35	8 (25%)	34 (58.6%)	
Preoperative CA19-9^a^			0.0011
≥529	16 (50%)	9 (15.5%)	
<529	16 (50%)	49 (84.5%)	
Preoperative CEA^a^			0.1616
≥2.4	19 (56.4%)	43 (74.1%)	
<2.4	13 (43.6%)	15 (25.9%)	
Preoperative Alb			0.381
≥3.5	25 (78.1%)	50 (86.2%)	
<3.5	7 (21.9%)	8 (13.8%)	
Preoperative WBC^a^			0.1868
≥5400	19 (59.4%)	25 (43.1%)	
<5400	13 (40.6%)	33 (56.9%)	
Preoperative lymphocyte^a^			0.3192
≥1023	26 (81.3%)	40 (69.0%)	
<1023	6 (18.7%)	18 (31.0%)	
Preoperative CRP^a^			0.7401
≥2.39	3 (9.4%)	8 (13.8%)	
<2.39	29 (90.6%)	50 (86.2%)	
mGPS			0.4168
1–2	8 (25%)	10 (17.2%)	
0	24 (75%)	48 (82.8%)	
Adjuvant therapy			0.4251
Yes	27 (84.4%)	44 (75.9%)	
No	5 (15.6%)	14 (24.1%)	

BMI body mass index, mGPS modified Glasgow prognostic score

^a^Cutoff value of tumor size, SPan-1, CA19-9, CEA, WBC, lymphocyte, and CRP was set by drawing ROC curve

^b^Recurrence within 1 year

^c^Recurrence at more than 1 year after surgery

**Table 3:** Multivariate analysis of factors affecting recurrence within 1 year after curative pancreatectomy for RPC

Predictor	OR	95% CI	*P* value
Histological grade	8.906	1.493–172.463	0.0129
Preoperative CA19-9	3.13	1.012–10.132	0.0477
Preoperative SPan-1	2.811	0.939–8.746	0.0647

*OR* odds ratio, *CI* confidence interval

### Recurrence patterns of RPC after surgery

Table 4 shows the comparison of postoperative recurrence of RPC. Among the 90 study patients, 60 (66.7%) experienced recurrence. The median OS of the patients with recurrence was 26.4 months, and their 5-year survival rate was 10%. Recurrence patterns were defined as the first recurrent locations. Distant metastasis included peritoneal dissemination, and local recurrence included regional lymph node and plexus nerve recurrence. Patterns of recurrence showed that 28 (46.7%) patients initially had distant metastasis and 32 (53.3%) patients initially had local recurrence. Among REC1, 22 (68.8%) patients had distant metastasis and 10 (31.2%) patients had local recurrence. On the contrary, among non-REC1, six (21.4%) patients had distant metastasis and 22 (78.6%) patients had local recurrence. REC1 experienced significantly more frequent distant metastasis than non-REC1.

**Table 4:** Comparison of postoperative recurrence patterns for RPC

	Total (*n* = 60)	REC1^a^ (*n* = 32)	Non-REC1^b^ (*n* = 28)	*P* value
Distant metastasis	28 (46.7%)	22 (68.8%)	6 (21.4%)	<0.001
Liver	6 (10%)	5 (15.6%)	1 (3.6%)	
Lung	7 (11.7%)	3 (9.4%)	4 (14.2%)	
Dissemination	9 (15%)	8 (25%)	1 (3.6%)	
Others	6 (10%)	6 (18.8%)	0 (0%)	
Local recurrence	32 (53.3%)	10 (31.2%)	22 (78.6%)	<0.001
Local	20 (33.3%)	6 (18.8%)	14 (50%)	
Lymph node	5 (8.3%)	1 (3%)	4 (14.3%)	
Plexus nerve	7 (11.7%)	3 (9.4%)	4 (14.3%)	

^a^Recurrence within 1 year

^b^Recurrence at more than 1 year after surgery

### Recurrence patterns and prognosis of the patients stratified by preoperative serum CA19-9 level and tumor diameter

Next, we focused on tumor diameter and preoperative serum CA19-9 level. We have encountered several patients in whom the preoperative serum CA19-9 level was very high despite small tumor diameter without distant metastasis; we suspect that such cases had distant metastasis undetectable by preoperative imaging modalities. We hypothesized that patients with small tumor diameter and high preoperative serum CA19-9 level experienced recurrence in the form of distant metastasis more frequently than the other patients. The study population was stratified into four groups according to tumor diameter and preoperative CA19-9 as follows (Table 5): group 1 (*n* = 5), CA19-9 level ≥529 U/mL and tumor size <25 mm; group 2 (*n* = 32), CA19-9 level <529 U/mL and tumor size <25 mm; group 3 (*n* = 20), CA19-9 level ≥529 U/mL and tumor size ≥25 mm; and group 4 (*n* = 33), CA19-9 level <529 U/mL and tumor size ≥25 mm. In group 1, all patients had recurrence, and four of the five patients (80%) experienced recurrence in the pattern of distant metastasis. In group 2, 16 of the 32 patients (50%) had recurrence, and seven of those 32 patients (21.9%) experienced recurrence in the pattern of distant metastasis. In group 3, 16 of the 20 patients (80%) had recurrence, and 11 of the 20 (55%) had distant metastasis. In group 4, 23 of the 33 patients (69.7%) had recurrence, and 10 of the 23 (30.3%) had distant metastasis. Further, regarding median OS, group 1 had a median OS of 12 months, group 2 did not reach the median, group 3 had a median OS of 27 months, and group 4 had a median OS of 45.3 months. The survival rate of group 1 was significantly poorer than that of group 2 (*P* = 0.0002; significant values of *P* < 0.01 was considered after Bonferroni correction).

**Table 5:** Recurrent patterns and prognoses of patients stratified by preoperative serum CA19-9 level and tumor diameter

Group	Number	CA19-9 ≥ 529	Tumor size ≥25 mm	Recurrence (*n* = 60)	Distant metastasis (*n* = 32)	Local recurrence (*n* = 28)	Median OS (months)	*P* value*
1	5	+	−	5 (100%)	4 (80.0%)	1 (20%)	12	
2	32	−	−	16 (50%)	7 (21.9%)	9 (28.1%)	Over 102.9	0.0002
3	20	+	+	16 (80%)	11 (55%)	5 (25%)	27	0.0485
4	33	−	+	23 (69.7%)	10 (30.3%)	13 (39.4%)	45.3	0.028

*Significant values of *P* < 0.01 was considered after Bonferroni correction

## Discussion

The present study indicated that the median OS of patients with RPC was 41.5 months, and 66.6% cases of patients with RPC experienced recurrence, including both recurrence within 1 year and recurrence beyond 1 year after surgery. The median OS of the recurrent cases among the RPC patients was 26.4 months, and the 5-year survival rate was 10%. Furthermore, the proportion of cases having recurrence within 1 year after curative resection for RPC was 35.6%, and the median OS was 14.2 months. These results suggest that even if the patients with RPC could be treated by curative surgery, more than half of them would experience recurrence, and their prognosis would be poor. Furthermore, independent risk factors for REC1 were histological grades G2–G4 and preoperative serum CA19-9 ≥ 529 U/mL. REC1 experienced recurrence in the pattern of more frequent distant metastasis, which might suggest that occult metastasis existed at the time of surgery. These results are crucial for the treatment strategy of RPC in the future.

The present study identified preoperative CA19-9 level ≥529 U/mL and histological grade G2-G4 as preoperative factors of recurrence within 1 year after curative resection for RPC. However, as it is usually difficult to diagnose the histological grade before surgery, the degree of differentiation is usually a post-resectional parameter. In fact, at our institution, the histological grade is not usually able to be diagnosed by endoscopic ultrasound (EUS)/fine needle aspiration (FNA) before surgery. Therefore, histological type cannot be considered as an appropriate preoperative factor. This suggests that the most appropriate preoperative independent factor is preoperative CA19-9 level ≥529 U/mL, excluding histological grade.

The CA19-9 tumor antigen was initially described by Koprowski et al. as a colorectal cancer marker [16] and is currently the most clinically useful marker of pancreatic cancer. Furthermore, CA19-9 is reliable as a possible prognostic marker for tumor resectability, recurrence, and survival [12, 17]. Berger et al. reported a longer median survival and greater percentage of patients surviving to 5 years among patients with either normal or undetectable preoperative levels of CA19-9 compared with patients having elevated levels [18]. Other investigators found that a postoperative decrease in CA19-9 and a postoperative CA19-9 value <200 U/mL were both significant predictors of survival in patients with pancreatic adenocarcinoma [4, 19]. Humphris et al. reported that normalization of CA19-9 within 6 months of resection was a prognostic factor [20]. On the other hand, it has been reported that only the preoperative CA19-9 level was associated with the prognosis or resectability of patients. Nakao et al. reported that the only preoperative predictor of survival time after surgery is a preoperative CA19-9 value ≥2000 U/mL [5]. Furthermore, other investigators have found that elevated preoperative CA19-9 levels were significantly associated with tumor unresectability, although the reported cutoff levels ranged from 100 to 350 U/mL [21–28]. In the present study, the AUC of the cutoff point of the CA19-9 value was 0.6815, and so it is difficult to say whether this AUC is the optimal value. Henceforth, the optimal cutoff value of CA19-9 should be evaluated by collecting a larger number of cases. Additionally, Turrini et al. suggested that the preoperative serum CA19-9 level by itself should not preclude surgery in patients who have undergone preoperative staging [29]. These investigators noted that even patients with very high values of preoperative CA19-9 may normalize their serum CA19-9 levels and have an overall survival equivalent to patients with normal preoperative serum CA19-9 levels. In the current study, we found that the group with higher CA 19-9 values tended to have poorer median OS. It is possible that preoperative serum CA19-9 level is associated with prognosis. But, careful evaluation is needed to determine whether the prognosis of this patient group was based on only the preoperative serum CA19-9 level.

We additionally examined the patterns of recurrence of RPC. The patients who experienced recurrence within 1 year had a greater amount of distant metastasis than local recurrence. These results might suggest that patients with early recurrence after curative surgery had minimally distant metastasis that was not detected via imaging modalities at the time of surgery, and their lesions did not visibly appear until after surgery.

Furthermore, we examined the recurrence patterns of each group stratified according to the combination of preoperative serum CA19-9 level and tumor size. The patients whose preoperative CA19-9 level was high despite smaller tumor diameter tended to have the poorest survival. From this result, it might also be speculated that patients whose preoperative CA19-9 level was high despite small tumor size had minimally distant metastasis before or at the time of surgery. It should be kept in mind that RPC patients with high preoperative serum CA19-9 levels have potentially distant metastasis. In particular, the possibility exists that patients whose preoperative CA19-9 level is high despite smaller tumor diameter have distant metastasis. Additionally, from the results of the present study, an appropriate cutoff value of preoperative serum CA19-9 value might be approximately 500 U/mL.

Chemotherapy for pancreatic cancer has recently been developed. A previous study reported that the median OS was 11.1 months after treatment with FOLFIRINOX for metastatic pancreatic cancer [30] and was 8.5 months after treatment with nab-paclitaxel plus gemcitabine [31]. The median OS of locally advanced pancreatic cancer is 24 months after first-line FOLFIRINOX with about 1/4 being resectable after this therapy [32]. Another study found that neoadjuvant chemotherapy contributed to high resection rates of local advanced pancreatic cancer [17]. Considering those reports, one option may be to administer preoperative chemotherapy for RPC patients who have high preoperative serum CA19-9 values, intending to exclude those patients who have the potential for early recurrence after surgery and to control minimally distant metastasis in those patients. It has recently been reported that staging laparoscopy in patients with local advanced pancreatic ductal adenocarcinoma is effective for selecting patients with occult distant organ metastasis [33]. To perform staging laparoscope for RPC patients having high preoperative CA19-9 values may make better patient selection for preoperative therapy.

In this study, we mainly focused on preoperative therapy. But, postoperative adjuvant therapy after curative resection has been showed to improve survival [34]. CONKO-001 showed treatment with gemcitabine for 6 months after complete resection of pancreatic cancer significant increased disease-free survival (DFS) and OS compared with observation alone. ESPAC-3 showed no significant difference in survival between adjuvant 5-fluorouracil/folinic acid and adjuvant gemcitabine. But, about 15% of patients may develop overt metastatic disease during postoperative recovery period; therefore, early initiation of adjuvant chemotherapy within 20 days after surgery has been shown to improve DFS and OS [35, 36]. These reports may indicate we should perform combination of preoperative therapy and postoperative early adjuvant therapy for RPC cases with CA19-9 ≥ 529. RCT trial (PREOPANC trial) as to preoperative radiochemotherapy versus immediate surgery for resectable and borderline resectable pancreatic cancer is conducted at the moment; the results will lead our studies to further directions [37].

The limitations of the present study were as follows. This was a retrospective study conducted at a single institution, and the sample size was small. Approximately 5–10% of the general population was Lewis antigen A- and B-negative, which meant that they did not synthesize CA19-9 antigen and would not have elevated levels, even with pancreatic cancer. In the present series, the data related to Lewis antigens A and B could not be included.

## Conclusions

Preoperative serum CA19-9 level ≥529 U/mL and histological grades G2–G4 were independent predictive factors for identifying recurrence within 1 year after pancreatectomy for RPC. Furthermore, REC1 had more frequent distant metastasis than non-REC1, which suggested that patients experiencing recurrence within 1 year had minimally distant metastasis. This might indicate the possibility that patients with high preoperative serum CA19-9 values and have potential distant metastasis despite suffering from RPC, especially, with small tumor, should receive combination of preoperative therapy and postoperative early therapy.

## Abbreviations

AUC: Area under the curve
BMI: Body mass index
BR-PC: Borderline resectable pancreatic cancer
CA19-9: Carbohydrate antigen 19-9
CEA: Carcinoembryonic antigen
CRP: C-reactive protein
DFS: Disease-free survival
mGPS: Modified Glasgow Prognostic Score
MST: Median survival time
NCCN: National Comprehensive Cancer Network
non-REC1: Patients did not experience recurrence within 1 year after curative resection for RPC
OS: Overall survival
OS: overall survival
REC1: Patients experienced recurrence within 1 year after curative resection for RPC
ROC: Receiver operating characteristic
RPC: Resectable pancreatic cancer
